# Ultrathin Bi_2_O_**2**_Se/Si Heterojunction Photodetector with Tunneling Oxide Passivation for Enhanced Optoelectronic Performance

**DOI:** 10.1021/acsami.5c03477

**Published:** 2025-04-23

**Authors:** Tzu-Pu Hung, Wei-Han Chen, Yi-Jyun Chen, Yu-Hao Tu, Zhi-Hao Huang, Yu-Lun Chueh, Chao-Hui Yeh, Chien-Wei Chen, Yang-Yu Jhang, Ying-Hao Chu, Cheng-Ying Chen

**Affiliations:** † Department of Materials Science and Engineering, National Tsing Hua University, Hsinchu 300044, Taiwan; ‡ Department of Chemical and Materials Engineering, Chang Gung University, Taoyuan 33302, Taiwan; § Department of Physics, National Sun Yat-Sen University, Kaohsiung 804201, Taiwan; ∥ Department of Materials Science and Engineering, Korea University, Seoul 02841, Republic of Korea; ⊥ Department of Electrical Engineering, National Tsing Hua University, Hsinchu 300044, Taiwan; # National Center for Instrumentation Research, National Applied Research Laboratories, Hsinchu 302058, Taiwan; ∇ Department of Optoelectronics and Materials Technology, National Taiwan Ocean University, Keelung 202301, Taiwan

**Keywords:** Bi_2_O_2_Se (bismuth oxyselenide), high responsivity, high on/off ratio, self-powered, photodetectors

## Abstract

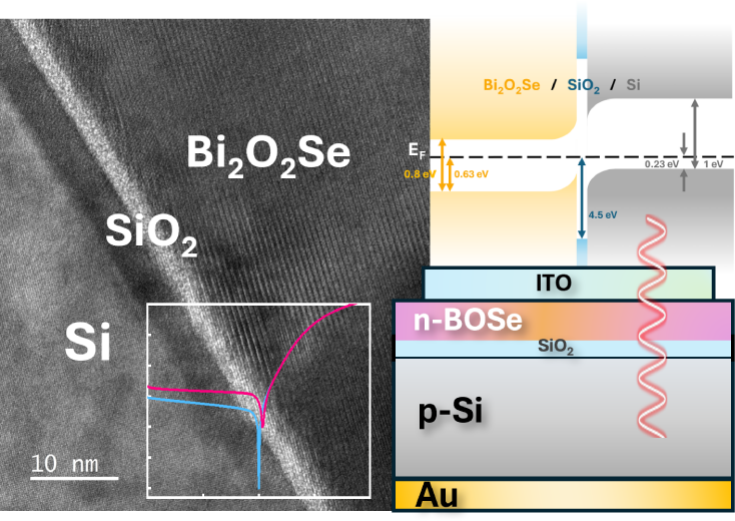

Two-dimensional (2D) materials have garnered significant attention for next-generation optoelectronic devices due to their exceptional physical properties. This study introduces a high-performance ultrathin Bi_2_O_2_Se/Si heterojunction photodetector with tunneling oxide passivation, fabricated using a transfer-free pulsed laser deposition method. The Bi_2_O_2_Se layer exhibits strong air stability and compatibility for practical applications. By incorporating a thin SiO_2_ tunneling layer, the heterostructure achieves a low dark current (∼22.3 nA/cm^2^), a high on/off ratio (∼8 × 10^6^), and a responsivity of 23.0 A/W. Compared to traditional CdS/Si devices, this photodetector demonstrates superior performance, including faster response time and higher stability. These findings underscore the potential of Bi_2_O_2_Se/Si heterostructures for advanced photonic and optoelectronic applications.

## Introduction

The rapid advancement of two-dimensional (2D) materials has sparked significant interest in their applications for next-generation electronic and optoelectronic devices, driven by their atomically thin structures^1^ and dangling-bond-free surfaces, which minimize carrier scattering and help maintain high carrier mobility even at reduced channel thicknesses^2,3^—achievements not easily met by conventional semiconductors. Among these 2D materials, bismuth oxyselenide (Bi_2_O_2_Se) stands out due to its impressive electron mobility,^4,5^ reaching up to 28,900 cm^2^ V^–1^ s^–1^ at 1.9 K,^6^ along with its inherent air stability^7^ and unique structure that accommodates a native high-k oxide layer, Bi_2_SeO_5_.^8^ These attributes position Bi_2_O_2_Se as a promising candidate for photodetectors,^9^ field-effect transistors,^6^ and thermoelectric applications.^10^

However, integrating Bi_2_O_2_Se with Si and enabling scalable device manufacturing remain challenging. Traditional fabrication methods, such as transfer techniques, yield high-quality single-crystal Bi_2_O_2_Se but fail to ensure high-quality fabrication with scalability for industrial production.^11^ Chemical vapor deposition, though more suited to large-scale synthesis, often produces polycrystalline films^12,13^ with grain boundaries that degrade electron mobility, limiting device performance. Moreover, the Bi_2_O_2_Se/Si interface may present defect states that introduce electronic instabilities, further hindering the development of Bi_2_O_2_Se/Si photonics devices.

To address these limitations, our work introduces a transfer-free approach to grow *c*-axis oriented Bi_2_O_2_Se directly on silicon using pulsed laser deposition (PLD), producing a high-quality heterojunction. To stabilize the interface and enhance optoelectronic performance, a thin tunneling oxide layer (SiO_2_) was introduced to passivate the heterointerface, reducing surface instabilities without compromising the electronic properties of Bi_2_O_2_Se. This heterostructure design leverages the natural n-type conductivity of Bi_2_O_2_Se, pairing it with a boron-doped p-type silicon substrate to form an effective photodiode structure, which demonstrates self-driven photodetection at room temperature with an impressive on/off ratio (∼8 × 10^6^), responsivity (23.0 A/W), and rapid response time of less than 1 ms. This innovative approach provides an opportunity of Bi_2_O_2_Se’s compatibility for practical applications and lays the groundwork for scalable, high-performance photodetectors, which have broad applications in integrated silicon photonics and beyond. Compared to other Bi_2_O_2_Se films directly grown on Si via CVD,^12,14−16^ PLD requires lower temperatures and a less stringent oxygen atmosphere. This allows for precise control over the SiO_2_ layer thickness through pretreatment without concerns about further oxidation caused by the environment. Compared to previous methods, this approach enhances the feasibility of vertically integrating Bi_2_O_2_Se with Si, offering greater potential for material applications.

## Materials and Methods

### Sample Preparation

**Figure 1:**
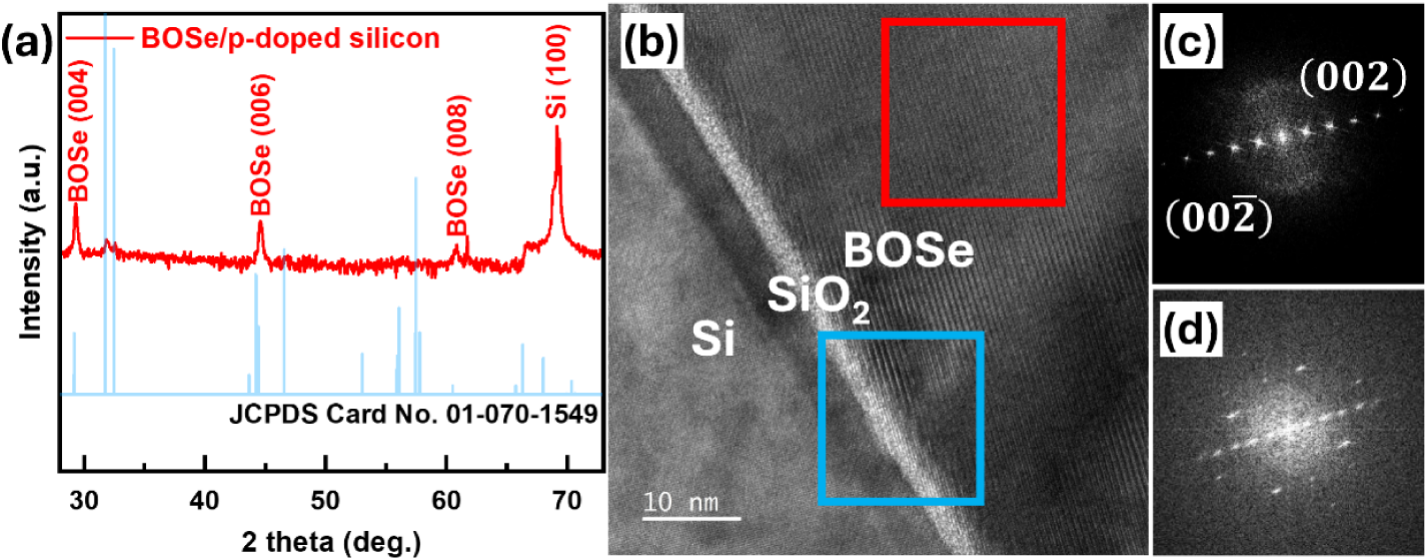
(a) XRD patterns of the Bi_2_O_2_Se/SiO_*x*_/p-Si heterostructure compared to the Bi_2_O_2_Se powder diffraction reference. (b) HRTEM image of the Bi_2_O_2_Se/SiO_*x*_/p-Si heterojunction, with SAED patterns corresponding to the regions outlined by the red (c) and blue (d) rectangles, respectively.

Si undergoes a series of pretreatments to ensure substrate quality before using as the deposition substrate. Given that the formation of SiO_2_ is nearly unavoidable at elevated temperatures and in an oxygen-rich environment, this pretreatment is essential. The SiO_2_ layer formed through this controlled process is characterized by its density, uniform thickness, and minimal layer depth (approximately 2–3 nm Figure 1b). This pretreatment ensures uniform substrate quality, thereby minimizing its impact on the growth characteristics of the thin film. The pretreatment procedure involves using Buffered Oxide Etch (BOE) to remove native SiO_2_ from the surface and establish a controlled chemical environment conducive to forming a uniform SiO_2_ layer. The detailed steps are as follows: the Si substrate is immersed in BOE for 30 s, followed by a rinse with deionized water. It is then placed in SC1 solution (NH_4_OH:H_2_O_2_:H_2_O = 1:1:5) at 70–75 °C for 30 s. This sequence is repeated three times to achieve a fully pretreated substrate.

Bi_2_O_2_Se thin films were synthesized via pulsed laser deposition (PLD) on pretreated silicon substrates using a high-purity (99.99%) Bi_2_O_2_Se target, commercially acquired in bulk from Ultimate Materials Technology Co., Ltd. Prior to deposition, the vacuum chamber was evacuated to a base pressure of 1 × 10^–6^ Torr, and subsequently filled with an oxygen atmosphere to a working pressure of 1 × 10^–1^ Torr. The substrate was heated to 425 °C during deposition, and upon completion, was cooled down at a controlled rate of 0.4 °C/s to room temperature.

Deposition was performed using a KrF excimer laser (λ = 248 nm; Lambda Physik, Coherent agent) operating at a repetition rate of 10 Hz with an energy density of 1 J/cm^2^. A total of 10,000 laser pulses were uniformly delivered to the Bi_2_O_2_Se target to ensure consistent material ablation and transfer onto the substrate. To minimize potential contamination, the cleaned substrate was immediately loaded into the deposition chamber after surface treatment.

Electrodes Au and ITO were deposited via RF sputtering under the following conditions. For Au electrodes, a sputtering power of 40 mW was applied for 300 s at a pressure of 1 × 10^–1^ Torr, resulting in a film thickness of approximately 100 nm. For ITO electrodes, sputtering was carried out with a power of 175 W for 1200 s under 3 × 10^–3^ Torr with an Ar gas flow rate of 30 sccm, yielding a thickness of approximately 200 nm. CdS was prepared following the method described by Chen et al.,^17^ where a chemical bath deposition (CBD) technique was used to grow a 50 nm thick CdS layer on the pretreated Si substrate. CBD was chosen rather than PLD for CdS growth, despite PLD offering better consistency with the growth method used for the previous material. This decision was made due to safety concerns associated with cadmium residues remaining in the deposition chamber after PLD, and the lack of a proper system to safely collect and dispose of these toxic materials. CBD, by contrast, provides a safer alternative with reliable reproducibility.

### X-ray Diffraction (XRD)

XRD measurements were performed at room temperature using a Bruker D2 Phaser diffractometer with Cu Kα radiation (λ = 1.5406 Å). Data were collected over a 2θ range of 20°–80° with an increment of 0.05°.

### Transmission Electron Microscope (TEM)

High-resolution TEM images were obtained using a JEOL JEM-F200 microscope operated at an accelerating voltage of 200 kV. Energy-dispersive X-ray spectroscopy analysis was conducted in STEM mode to determine the elemental composition of the samples.

### X-ray Photoelectron Spectroscopy (XPS)

XPS measurements with high spatial resolution were conducted at the TLS09A1, NSRRC in Hsinchu, Taiwan, using photon energies of 620 eV. All measurements were performed at room temperature.

### Optoelectronic Measurements

The optoelectronic measurements were conducted using the Keysight B1500A Semiconductor Device Parameter Analyzer connected to two electrodes. Monochromatic laser beams of varying frequencies and energies illuminated the device at specific points.

## Results and Discussion

The direct growth of the thin film on Si is challenging due to the significant lattice mismatch at the interface, which often leads to defects such as dislocations or poor crystallinity.^18^ Thus, structural analysis of the Bi_2_O_2_Se synthesized directly on Si was implemented. In Figure 1a, the X-ray diffraction (XRD) pattern demonstrates that the Bi_2_O_2_Se thin film exhibits a pronounced (00l) preferred orientation, with only the substrate’s signal detected. This observation suggests that the film predominantly grows along the (00l) direction. The small peak observed around 33° 2θ in Figure 1a may suggest the presence of a minor degree of polycrystallinity near the interface. Since the Bi_2_O_2_Se film is directly grown on SiO_2_, which presents a considerable lattice mismatch, it is possible that a thin polycrystalline layer forms at the interface. This layer may serve as a buffer to accommodate the strain and facilitate subsequent epitaxial growth. The shoulder appearing near 66° in the XRD pattern is attributed to the residual Kα_2_ component due to incomplete filtering by the Ni filter in the X-ray diffractometer, which does not indicate the presence of secondary phases or microstructural features in the sample.

However, this alone is not sufficient to fully confirm the result; therefore, high-resolution transmission electron microscopy (HRTEM) was employed for further verification. Figure 1b shows HRTEM results confirming the film’s crystallinity, thereby verifying that a highly ordered Bi_2_O_2_Se layer has indeed been achieved. This result also matches the selected area electron diffraction (SEAD) in Figure 1c,d, which presents the heterojunction of Bi_2_O_2_Se/SiO_*x*_/p-Si observed through HRTEM. The clear interfaces between Si/SiO_*x*_ and SiO_*x*_/Bi_2_O_2_Se can be seen in Figure 1b. Notably, it is observed that the *c*-axis of Bi_2_O_2_Se is tilted relative to the Si (100) out-of-plane orientation, a phenomenon attributed to the substantial lattice mismatch between the two materials.^19^ This high degree of crystallinity highlights the effectiveness of our fabrication method. The consistency between the XRD and HRTEM results confirms the thin film quality, demonstrating that our fabrication has overcome the challenges of integrating two-dimensional materials with silicon.

**Figure 2:**
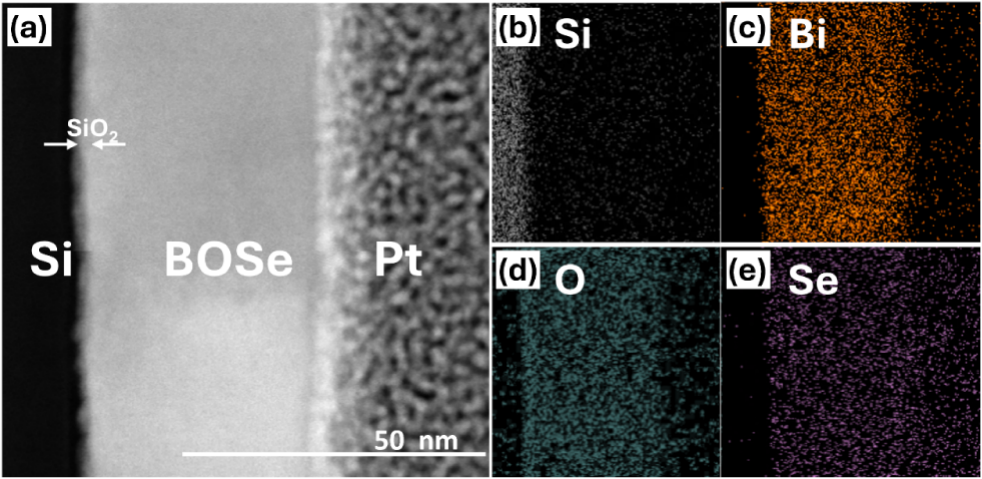
(a) TEM image of the Bi_2_O_2_Se/SiO_*x*_/Si heterojunction. (b–e) EDS elemental mapping derived from the area in (a), illustrating the distributions and relative proportions of Si, Bi, O, and Se.

To examine the stoichiometric ratios of Bi–O–Se compounds, the Energy Dispersive X-ray Spectroscopy (EDS) mapping was employed. Figure 2a presents the electron microscope image, in which the Pt layer was deposited during the sample preparation process using a Focused Ion Beam (FIB) to serve as a protective layer, preventing damage to the sample. While the corresponding EDS mappings for Bi, O, and Se are shown in Figure 2c–e. From the EDS mapping in Figure 2c–e, it is evident that our thin film exhibits uniformity. Additionally, Table S1 reveals that the Bi:O:Se ratio provided by EDS is 2:2:0.8, which closely matches the stoichiometric ratio of Bi_2_O_2_Se. The selenium deficiency leads to the formation of selenium vacancies (V_Se_), which create donor states, resulting in the n-type semiconductor behavior of Bi_2_O_2_Se.^20^ Moreover, the part of selenium deficiency might also result from damage incurred during the FIB process, leading to the loss of weaker bonded [Se]^2–^ layers.^21^ The consistent stoichiometry and uniformity observed in both surface and depth analyses suggest that the growth conditions were well-controlled, minimizing phase separation or stoichiometric deviation, which are common issues in the fabrication of complex oxide thin films.

**Figure 3:**
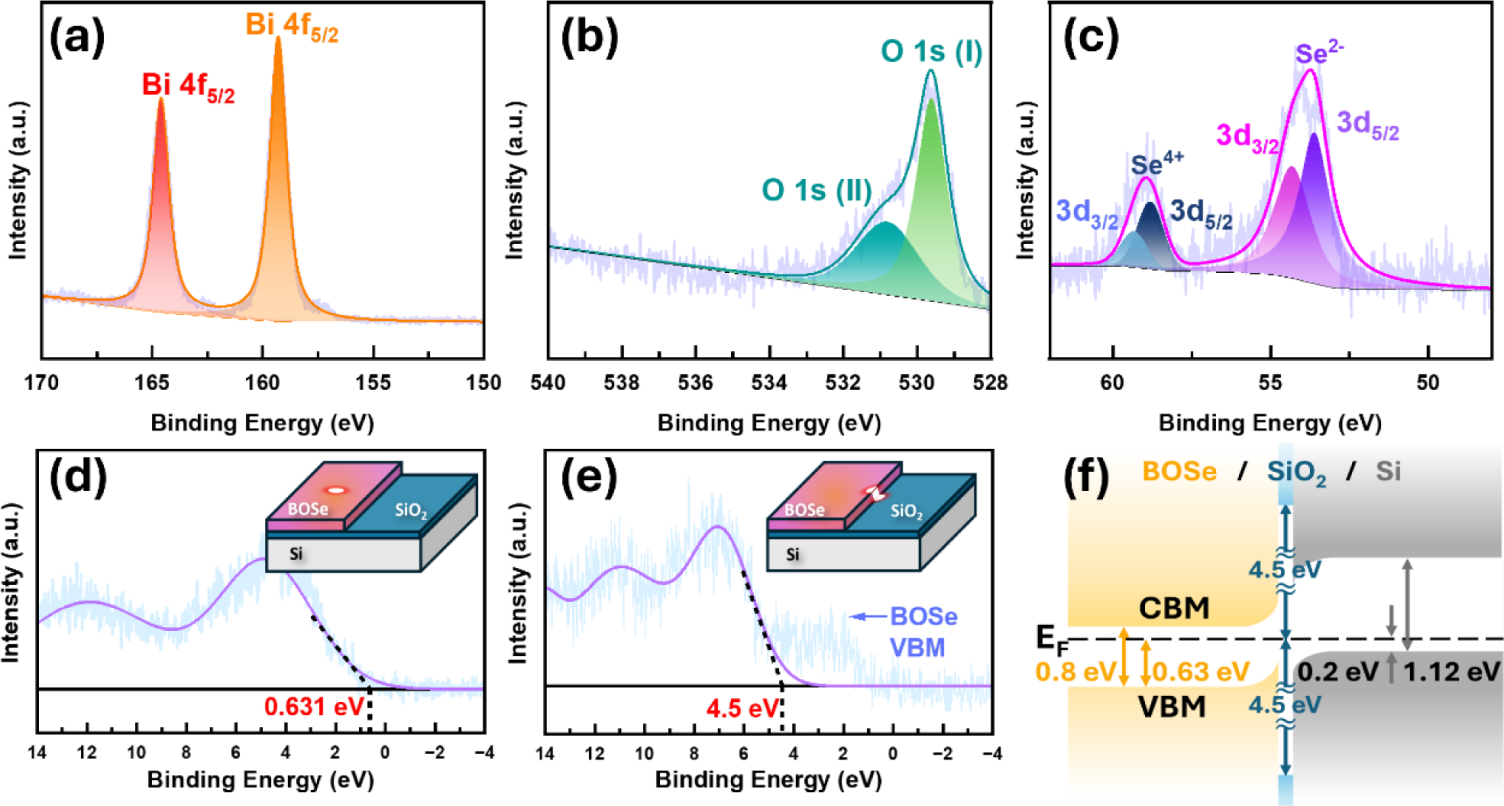
XPS spectra of (a) Bi 4f, (b) O 1s, and (c) Se 3d of Bi_2_O_2_Se on Si. XPS valence band maximum analysis of (d) Bi_2_O_2_Se and (e) SiO_2_. (f) Band alignment of the Bi_2_O_2_Se/SiO_2_/Si heterojunction.

Building on the above-mentioned analysis, X-ray photoelectron spectroscopy (XPS) provides further insights into the elemental composition and chemical states. To avoid detecting signals from SiO_2_, a 20 nm Bi_2_O_2_Se layer was grown, twice thicker than the XPS penetration depth. Figure 3a shows the XPS spectrum with Bi 4f appearing at 164.6 and 159.3 eV, which matches Bi 4f_5/2_ and Bi 4f_7/2_. O 1s(I) (529.6 eV), Se 3d_3/2_ (54.3 eV) and Se 3d_5/2_ (53.6 eV) are shown in Figure 3b,c as well. The peaks corresponding to O 1s(II) (530.8 eV) in Figure 3b and Se 3d_3/2_ (59.32 eV) and Se 3d_5/2_ (58.8 eV), representing Se^4+^, in Figure 3c are indicative of the spontaneous oxidation state Bi_2_SeO_5_, as reported in other studies on Bi_2_O_2_Se.^22,23^ This oxide layer is exceedingly thin, so no corresponding signals appear in the XRD in Figure 1a, EDS in Figure 2, and Raman in Figure S1. However, the chemical states within a few atomic layers can be significantly revealed through high-resolution XPS at the National Synchrotron Radiation Research Center (NSRRC), Taiwan. This ultrathin oxide layer (Bi_2_SeO_5_,) also helps reduce the density of dangling bond states on the Bi_2_O_2_Se surface, which plays the same role as SiO_2_ on Si, which significantly decreases dangling bond states and enhances the stability of its electrical performance. Besides the chemical states, XPS provides insights into the valence band maximum (VBM) analysis; utilizing XPS, measurements were conducted at various locations within the heterostructure. As shown in Figure 3d, the energy difference between the VBM and the Fermi level (*E*_*F*_) on Bi_2_O_2_Se is measured to be 0.63 eV, indicating that the Fermi level of Bi_2_O_2_Se is closer to the conduction band minimum (CBM), i.e., 0.17 eV difference, confirming the n-type semiconductor behavior of Bi_2_O_2_Se. At the interface between Bi_2_O_2_Se and SiO_2_, illustrated in Figure 3e, a separation of 4.5 eV is observed, consistent with the SiO_2_ values reported in the previous studies.^24,25^ In addition to the XPS spot covering the area between Bi_2_O_2_Se and SiO_2_, as shown in the inset of Figure 3e, there is the second VBM drop in Figure 3e corresponds to the identified Bi_2_O_2_Se energy gap of 0.8 eV in Figure 3d. The Fermi level (*E*_*F*_) position in Si can be determined by^26^
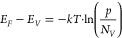
1where *p* is the carrier concentration of dopant 5 × 10^15^*cm*^–3^, *k* is the Boltzmann constant, *N*_*V*_ is the valence band density of states, and *T* is the temperature. The distance between the Fermi level and VBM is about 0.2 eV. Combining the above, the resulting heterojunction band alignment was plotted, as shown in Figure 3f. This analysis reveals that the heterostructure is a type-II heterojunction with ultrathin passivation oxide tunneling barrier, which allows for efficient separation of photogenerated electron–hole pairs, reducing carrier recombination rates and facilitating longer carrier lifetimes. Such properties are particularly advantageous for optoelectronic applications, as they contribute to low dark current, which enhances signal-to-noise ratio, and high responsivity, ensuring superior sensitivity to incident light. These energy band structures have the capability to enhance the performance of our device and position it as a promising candidate for advanced photodetectors and other optoelectronic applications. A detailed discussion of the device performance will be presented in subsequent sections.

The photodiode devices featuring appropriately matched work function metals were fabricated to demonstrate the superior optoelectronic performance of the Bi_2_O_2_Se/SiO_2_/Si heterojunction. To optimize the photoelectronic performance of the heterostructure, it is crucial to minimize the influence of the electrodes on electron transport. Gold (Au) was selected for the bottom electrode because its work function (5.1 eV) closely aligns with that of p-Si, thereby reducing band bending and mitigating the formation of a Schottky barrier. For the top electrode on the Bi_2_O_2_Se layer, Indium Tin Oxide (ITO), with a work function of 4.7 eV, was chosen, as it is well suited for n-type semiconductors. Moreover, ITO offers excellent optical transparency, enabling the most visible light to illuminate the diode structure. Importantly, ITO can be deposited without high-temperature processing, preserving the thin film’s quality. Beyond the previously discussed band structure advantages, additional studies confirm that Bi_2_O_2_Se is a promising material for optoelectronic device fabrication. Figure S2 provides a detailed representation of the energy band information for all materials involved in the device.

**Figure 4:**
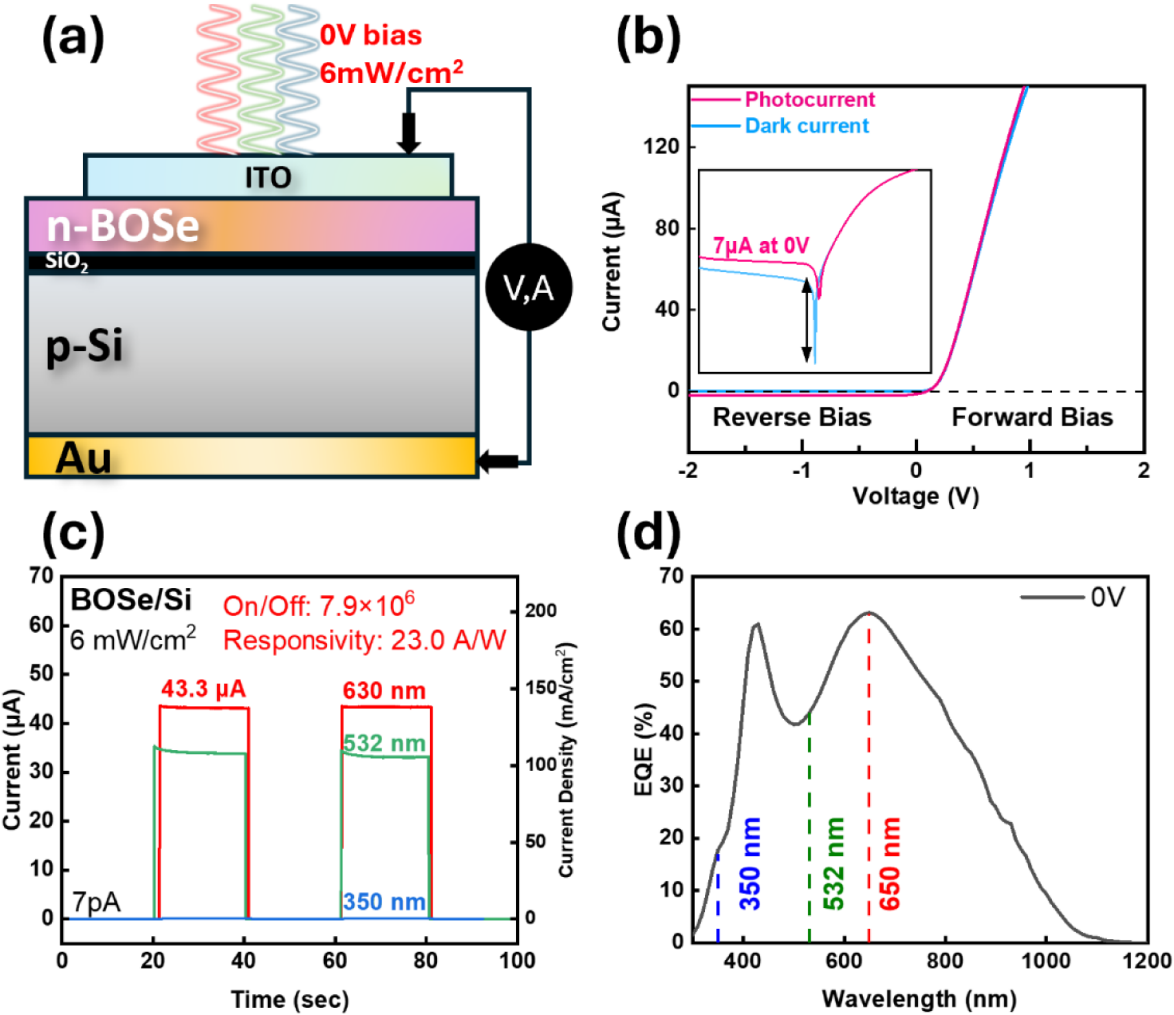
(a) Structure of the Bi_2_O_2_Se/Si heterojunction with top ITO electrode and back Au electrode. (b) IV measurements before and after light illumination, presented with a linear scale and a logarithmic scale (in the inset). (c) Photoelectric properties with three kinds of single wavelength laser illumination without voltage bias. (d) EQE measurement from 300 nm to 1200 nm under 0 V bias.

As shown in Figure 4a, electrodes were deposited on both the top and bottom of the device to facilitate measurements. The thickness of the Bi_2_O_2_Se layer is ∼13 nm. Regarding thickness optimization, Figure S3 demonstrates that the 13 nm Bi_2_O_2_Se layer yields the highest photoresponse (43.3 μA), indicating it as the optimal thickness. In general, thinner films outperform thicker ones due to reduced photon competition with the underlying silicon. However, the film should not be too thin, as excessively thin layers may suffer from nonuniform growth and insufficient carrier mobility. The I–V curve of the Bi_2_O_2_Se/Si heterojunction, presented in Figure 4b, demonstrates its pn diode rectifying characteristics with full-spectrum light with an intensity of 2 mW/cm^2^ irradiated onto it. Additionally, the I–V curve in the log scale (the inset of Figure 4b) can be observed that the most significant on/off ratio occurs at 0 V bias, and it also shows a comparative performance with the photocurrent under applied bias. The equation below applies the WKB (Wentzel–Kramers–Brillouin) approximation to calculate the tunneling probability (*T*) of an electron or hole, with energy *E* and effective mass *m*, traversing an oxide potential barrier *V(x)* of finite thickness *d* (2–3 nm):
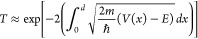
2

When the diode built-in potential is significant (as shown in Figure 3f) and oxide thickness *d* is ultrathin (2–3 nm), the exponent approaches zero, and the tunneling probability *T* remains close to 100%. Consequently, the SiO_2_ layer has minimal impact on the photocurrent performance of the device. Furthermore, previous studies have noted that introducing a tunneling SiO_2_ layer can reduce dark current, enhancing device stability,^27,28^ and further providing a superior on/off ratio similar to our observation, as shown in the inset of Figure 4b.

Figure 4c illustrates the photoresponse of the Bi_2_O_2_Se/Si heterostructure under illumination with three different wavelengths—630 nm (red), 532 nm (green), and 350 nm (UV)—at the same intensity of 6 mW/cm^2^ and without voltage bias. Significant responses were observed for the 630 and 532 nm wavelengths, while the response in the UV region was negligible. The ultrathin SiO_2_ tunneling layer contributed to the device’s extremely low dark current (∼22.3 nA/cm^2^), enhancing its performance. Under 630 nm red light, the device achieved optimal performance, with an impressive on/off ratio of 7.88 × 10^6^ and a responsivity of 23.0 A/W.
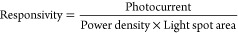
3
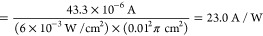


Furthermore, the response time was exceptionally fast, with both rise time (τ_*r*_) and decay time (τ_*d*_) measured at less than 1 ms, defined here as the time taken for the signal to transition between 10% and 90% of its maximum value. Notably, the measured ∼1 ms response time was a limitation of the measurement setup rather than the actual capability of the device. To confirm reproducibility and stability, additional devices were fabricated under the same conditions. As shown in Figures S4 and S5, the devices exhibit consistent photoresponse and stable performance over repeated on–off illumination cycles, verifying the reliability of the fabrication process.

To comprehensively understand the wavelength-dependent photoresponse of our devices at 0 V bias, External Quantum Efficiency (EQE) measurements were conducted. The EQE curve (Figure 4d) illustrates the photoelectric conversion efficiency over the wavelength range of 300 to 1200 nm. The reduced photoelectric response below 400 nm is attributed to the optical absorption of the top ITO thin film. Additionally, optical interference effects caused by the thickness of the ITO layer result in significant optical reflection near 500 nm, leading to a decrease in EQE. The curve also reveals that the longest wavelength for the photoelectric response is 1100 nm, corresponding to the bandgap of silicon (1.1 eV) rather than Bi_2_O_2_Se (0.8 eV). This observation is expected, as the Bi_2_O_2_Se layer, being only ∼13 nm thick, contributes minimally to photon absorption. However, this does not imply that Bi_2_O_2_Se is unimportant in the device. On the contrary, the ∼13 nm n-type Bi_2_O_2_Se, combined with p-type Si, forms a built-in potential (as shown in Figure 3f), which acts as the driving force for photocarriers. This built-in potential is essential for achieving the observed photoelectric conversion efficiencies at 0 V bias. Moreover, the EQE curve helps explain why the device, as shown in Figure 4c, demonstrated optimal performance under 630 nm red light, achieving an impressive responsivity of 23.0 A/W.

**Figure 5:**
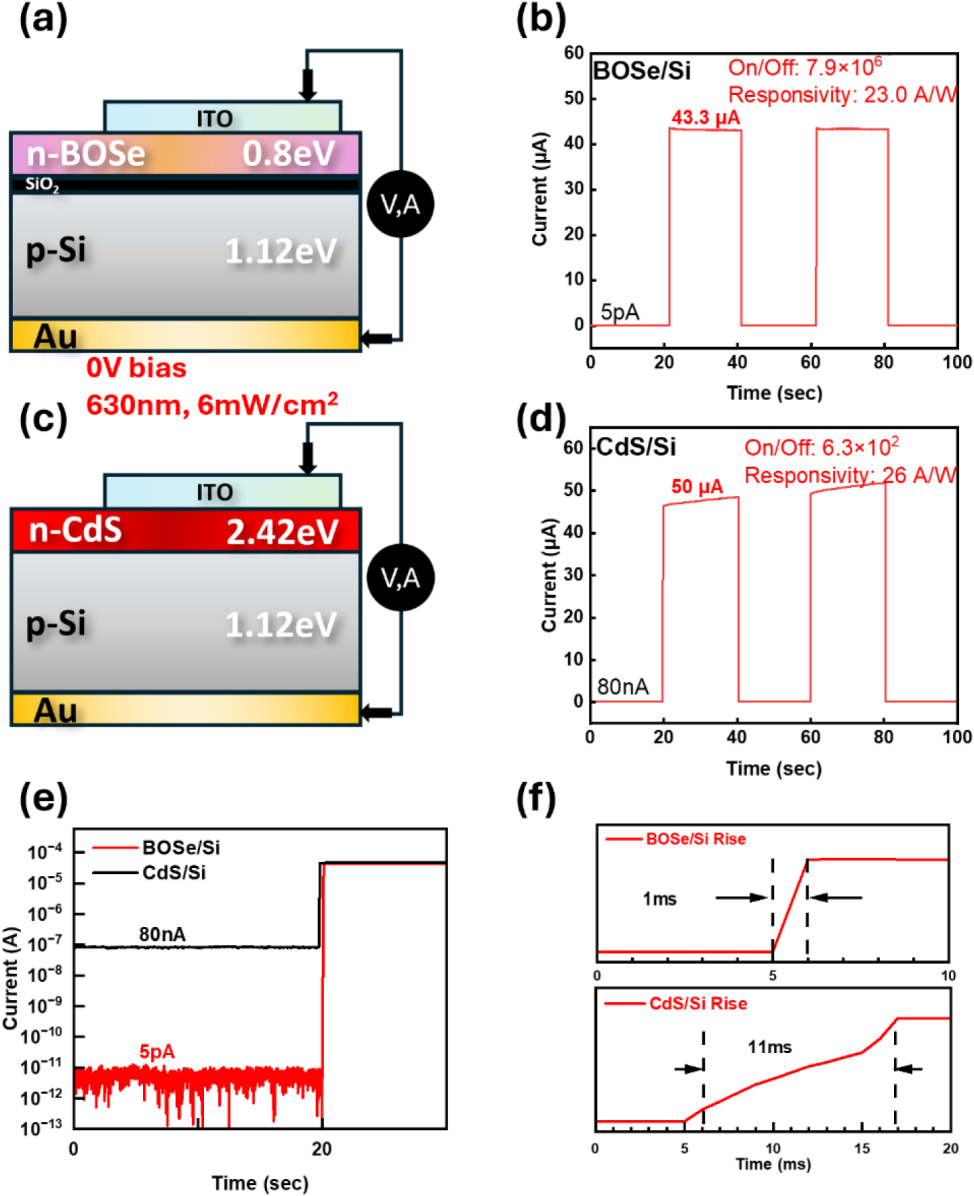
(a)Measurement and the (b) result of Bi_2_O_2_Se/Si heterojunction. (c) The structure that Bi_2_O_2_Se is replaced with CdS and the corresponding bandgap. (d) Result of CdS/Si heterojunction. All these measurements are operated under 0 V bias with the irradiation of 6 mW/cm^2^ 630 nm red light. (e) Comparison of dark current and (f) response time between Bi_2_O_2_Se/Si and CdS/Si.

To further investigate the contribution of Bi_2_O_2_Se in this heterostructure optoelectronic device (Figure 5a), a comparison device using the conventional n-type semiconductor material CdS was fabricated for evaluation, as shown in Figure 5c. CdS was chosen for comparison primarily because it has been widely utilized in optoelectronic applications. Additionally, CdS can be rapidly synthesized with high stability using a CBD method, ensuring consistent material quality and eliminating concerns regarding material variability in comparative studies. Furthermore, the bandgap of CdS is 2.4 eV, the photoresponse comparison experiment was conducted at 630 nm, ensuring that photocarriers are predominantly generated within the Si layer. In this setup, the n-type materials (either CdS or Bi_2_O_2_Se) primarily function to establish the built-in electric field and facilitate the collection of photogenerated electrons. The comparison measurement results are shown in Figure 5d, under 6 mW/cm^2^ 630 nm red light illumination at 0 V bias, the choice of CdS thickness was based on preliminary measurements, where a 20 nm CdS film showed negligible photoresponse. Therefore, we selected a thicker CdS layer that produced a comparable responsivity to our Bi_2_O_2_Se/Si device, to enable a more meaningful comparison. The responsivity in the CdS/Si heterostructure remains comparable to that of the Bi_2_O_2_Se/Si device. However, under continuous light illumination, the photocurrent in the CdS/Si device gradually increases, a phenomenon commonly observed in semiconductor thin films with higher defect densities. Additionally, in the CdS/Si device, the dark current increases by 4 orders of magnitude compared to the Bi_2_O_2_Se/Si device (10^–12^ A), reaching 10^–8^ A, leading to a significant reduction in the on/off ratio, as shown in Figure 5e. This difference could be attributed to the higher defect density at the CdS/Si interface, whereas the Bi_2_O_2_Se/Si device benefits from tunneling oxide passivation, which significantly reduces the dark current. Figure S6 compares the dark current in CdS/Si and CdS/SiO_2_/Si devices. Introducing a 3 nm SiO_2_ layer reduced the dark current from 80 nA to 50 nA. However, Bi_2_O_2_Se-based devices still exhibited lower dark current, highlighting the importance of material-dependent interface engineering. Direct comparison in Bi_2_O_2_Se devices (without SiO_2_) is limited due to unavoidable native oxide formation during PLD. Moreover, when CdS replaces Bi_2_O_2_Se, the response time increases from 1 to 10 ms, as shown in Figure 5f. This change can be attributed to the mobility difference between Bi_2_O_2_Se and CdS.

Based on these measurements, we conclude that the Bi_2_O_2_Se/Si heterostructure benefits from the introduction of the passivation SiO_2_ layer. Additionally, the anisotropic conductivity of Bi_2_O_2_Se, where conduction is lower along the *c*-axis and higher along the ab-plane^26,27^, further contributing to the low dark current at zero bias, enhancing the on/off ratio. The high carrier mobility of Bi_2_O_2_Se, as reported in previous studies,^6^ also accounts for the rapid response speed observed. These properties are further confirmed through comparison with the CdS replacement, which reveals distinct trends and underscores the role of Si in the heterostructure as the primary contributor to photocurrent generation and responsivity. A detailed comparison of the experimental data on dark current, on/off ratio, response time, and responsivity, as related to Figures 4 and 5, is provided in Table S2.

For a better understanding of the performance of our device, the comparison sheet of 2D materials/Si heterojunction-based photodetectors is listed in Table 1. Notably, our structure demonstrates excellent responsivity under zero bias condition, which is particularly meaningful for low-power optoelectronic applications. Additionally, the absorption spectrum of our structure is more inclined toward the red-light region, showing superior responsiveness compared to devices of similar performance levels. Regarding the response time, a direct comparison is challenging. Due to a data point interval of 1 ms, the exact response time cannot be determined, but it is confirmed to be less than 1 ms. However, numerous studies based on 2D materials have consistently reported response times within the microsecond range, which reflects the general potential of 2D materials/Si heterojunctions for high-speed operation. We believe that our device also shares this potential, supported by the high carrier mobility characteristics of 2D materials. And the favorable band alignment between Bi_2_O_2_Se and Si, which reduces the energy barrier at the interface and facilitates efficient carrier transport. This allows a simple p–n junction configuration to achieve effective and rapid photoresponse without the need for complex interface engineering.

**Table 1:** Comparisons of 2D Materials/Si Heterojunction Devices

Materials	Conditions	R (A/W)	Time (Rise/Decay)	Ref.
Bi_2_O_2_Se/SiO_2_/Si	630 nm/0 V	23.0	<1 ms^a^	This Work
WS_2_/Si	365 nm/0 V	0.004	1.1 μs	(29)
MoTe_2_/Si	700 nm/0 V	0.26	5 ns/8 ns	(30)
MoS_2_/Si	808 nm/0 V	0.3	3 μs/40 μs	(31)
Gr/n-Si	890 nm/0 V	0.73	0.32 ms/0.75 ms	(32)
Bi_2_Se_3_/Si	808 nm/0 V	2.6	2.5 μs/5.5 μs	(33)
Bi_2_Se_3_/Si	808 nm/–1 V	24.28	2.5 μs/5.5 μs	(33)
Mg_2_Si/Si	1064 nm/–1 V	0.06	1.72 ms/1.61 ms	(34)
MoS_2_/Si	650 nm/–2 V	11.9	31 μs/72 μs	(35)
MoS_2_/Si	550 nm/–5 V	9	9 μs/7 μs	(36)
Gr/SiO_2_/p-Si	633 nm/–5 V	1.2	40 ns/100 ns	(37)
Gr/SiO_2_/n-Si	633 nm/–5 V	0.45	40 ns/100 ns	(37)
WS_2_/Si	650 nm/–5 V	5.7	670 μs/998 μs	(38)

a Limited by the measurement capabilities of the equipment, whose data interval is 1 ms.

## Conclusion

In this study, we successfully demonstrated the direct growth of ultrathin Bi_2_O_2_Se on p-type silicon substrates using a transfer-free pulsed laser deposition method. The introduction of a 3 nm SiO_2_ tunneling oxide layer effectively passivated the heterointerface, reducing surface instabilities and enhancing the optoelectronic performance of the Bi_2_O_2_Se/Si heterostructure. The resulting photodetector exhibited exceptional performance metrics, including a low dark current (∼22.3 nA/cm^2^), a high on/off ratio (∼8 × 10^6^), and a responsivity of 23.0 A/W under zero bias. These attributes surpass traditional CdS/Si devices in both response speed and the saturation behavior.

Material analyses confirmed the high crystallinity, uniform stoichiometry, and excellent electronic properties of the heterostructure, validating the efficacy of the fabrication process. The device’s scalability, compatibility with CMOS processes, and enhanced performance metrics establish it as a strong candidate for next-generation photodetectors and optoelectronic systems. This work provides a significant step toward the integration of two-dimensional materials into silicon photonics, offering new opportunities for the development of high-performance and scalable optoelectronic devices.

## Supporting Information Available

The detailed information on EDS line scan and Raman spectrum of our as-grown thin films; the energy band information on all materials used in the Bi_2_O_2_Se device from top to bottom; the photoresponse under different Bi_2_O_2_Se thicknesses; the reproducibility/stability tests of the additional Bi_2_O_2_Se devices; comparison of dark current with and without 3 nm SiO_2_ in the CdS devices; the detailed experimental data comparison of Figures 4 and 5 (PDF)
