# Supplementary material for: Ultrathin Bi2O2Se/Si Heterojunction Photodetector with Tunneling Oxide Passivation for Enhanced Optoelectronic Performance
Source: ACS Appl Mater Interfaces. 2025 Apr 23;17(18):26931–9. doi: 10.1021/acsami.5c03477 (PMC12067371; doi:10.1021/acsami.5c03477)
Supplement: Supplementary file 1 — am5c03477_si_001.pdf [file am5c03477_si_001.pdf]

Supporting Information

Title: Ultra-Thin Bi_2O_2Se/Si Heterojunction Photodetector with Tunneling Oxide Passivation for Enhanced Optoelectronic Performance

Tzu-Pu Hung[1], Wei-Han Chen[1], Yi-Jyun Chen[1], Yu-Hao Tu[1], Zhi-Hao Huang[2], Yu-Lun Chueh[1,3,4], Chao-Hui Yeh[5], Chien-Wei Chen[6], Yang-Yu Jhang[6], Ying-Hao Chu[1*], Cheng-Ying Chen[7*]

[1]Department of Materials Science and Engineering, National Tsing Hua University, Hsinchu 300044, Taiwan

[2]Department of Chemical and Materials Engineering, Chang Gung University, Taoyuan 33302, Taiwan

[3]Department of Physics, National Sun Yat-Sen University, Kaohsiung 804, Taiwan.

[4]Department of Materials Science and Engineering, Korea University, Seoul 02841, Republic of Korea.

[5]Department of Electrical Engineering, National Tsing Hua University, Hsinchu 300044, Taiwan

[6]National Center for Instrumentation Research, National Applied Research Laboratories, Hsinchu 302058, Taiwan

[7]Department of Optoelectronics and Materials Technology, National Taiwan Ocean University, Keelung 202301, Taiwan

*Correspondence to: chen.chengying.cyc@gmail.com (C.-Y. Chen);

yhchu@mx.nthu.edu.tw (Y.-H. Chu)

Table S1 reveals that the Bi:O:Se ratio provided by EDS is 2:2:0.8, which closely matches the stoichiometric ratio of Bi_2O_2Se.

Table S1. Detailed information of EDS line scan

Elements	Line Type	k factor	Wt %	Atomic %
Bi	L Series	3.201	74.6	30.82
O	K Series	1.455	74.6	30.36
Se	K Series	2.223	11.09	12.12
Si	K Series	1.000	8.69	26.70

Figure S1 presents the Raman spectrum of our 100 nm-thick sample. It shows a distinct characteristic peak at 160 cm^{-1}, corresponding to the A_1g mode of Bi_2O_2Se, while no signals corresponding to Bi_2SeO_5 were observed [1].

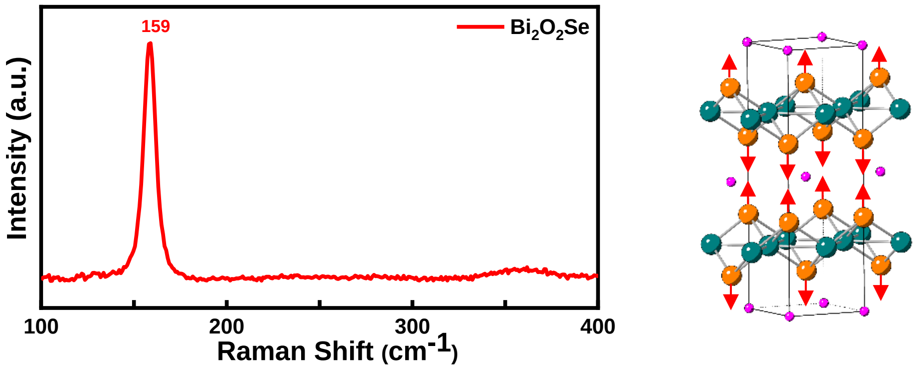

Figure S1. The Raman spectrum of our Bi_2O_2Se/Si sample.

Photodiode devices with well-matched metal work functions were fabricated to demonstrate the superior optoelectronic performance of the $Bi_2O_2Se/SiO_2/Si$ heterojunction. To optimize performance, it is important to minimize the impact of electrodes on electron transport. Gold (Au, work function 5.1 eV) was used as the bottom contact due to its alignment with p-Si, reducing band bending and Schottky barrier formation. Indium Tin Oxide (ITO, work function 4.7 eV) was chosen as the top electrode for its suitability with n-type semiconductors, excellent optical transparency,

and low-temperature process compatibility. The energy band alignment of all materials is illustrated in **Figure S2**.

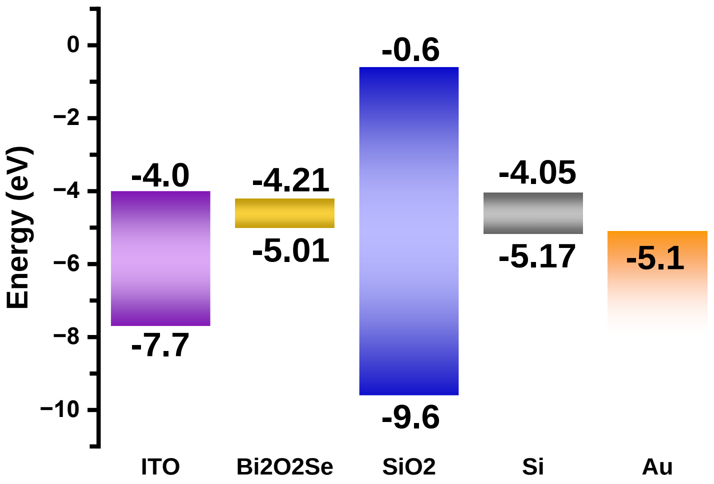

Figure S2. The energy band information of all materials used in the device from top to bottom.

Figure S3 demonstrates an optimal thickness range for achieving superior device performance, where thinner films generally result in better efficiency. This is primarily because the main light-absorbing material in the device is silicon. Given that Bi_2O_2Se also absorbs light within a similar spectral range due to its bandgap, a thicker Bi_2O_2Se layer may compete with silicon for photon absorption, thereby decreasing overall efficiency. However, the Bi_2O_2Se layer should not be made excessively thin, for two key reasons. Firstly, extremely thin Bi_2O_2Se films directly grown on Si tend to suffer from non-uniformity, which adversely impacts device uniformity and performance. Secondly, thicker Bi_2O_2Se films possess higher carrier mobility than purely two-dimensional structures. This observation aligns with the findings of Huang *et al*[2]., who reported that bulk Bi_2O_2Se structures exhibit notably improved transport characteristics.

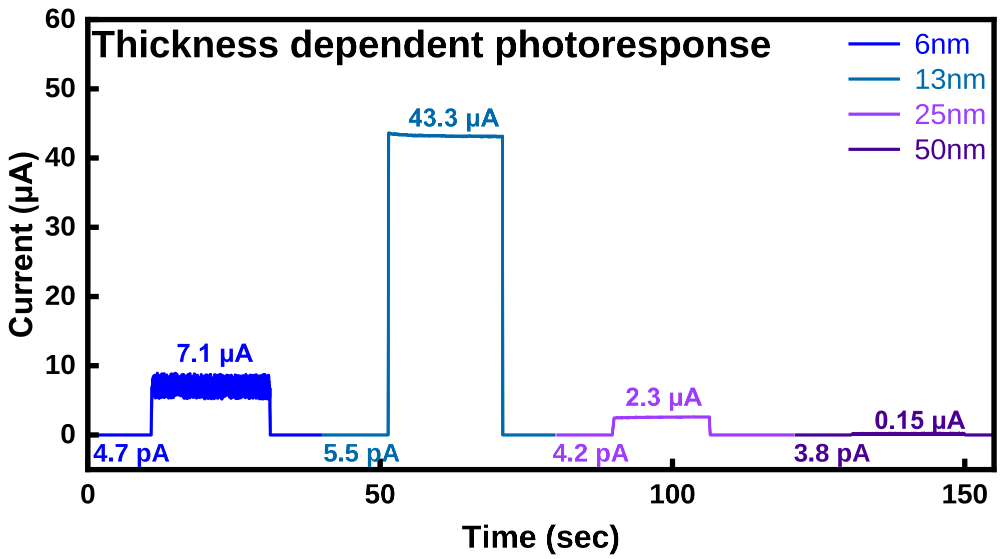

Figure S3. Photoresponse under different Bi_2O_2Se thicknesses

To ensure the reproducibility of our experimental results, additional devices were fabricated using the same parameters as those used in this study, as shown in **Figure S4**. All the samples consist of 13 nm Bi_2O_2Se / 3 nm SiO_2 / p-type Si heterostructures. These samples also underwent optoelectronic characterization, operated under 0V bias with the irradiation of 6 mW/cm^2 630nm red light. As can be seen, the performance metrics are consistent with those reported in the main manuscript, confirming the reproducibility of our fabrication process.

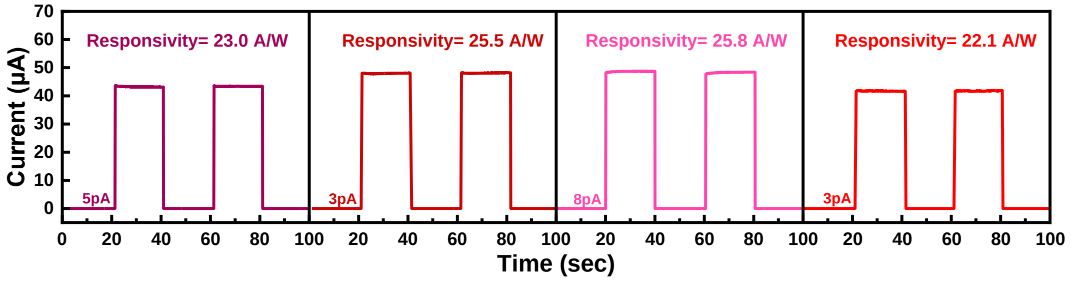

Figure S4. I-t curve of the reproducible test

In **Figure S5**, during the stability test, the device underwent 100 on–off illumination cycles, each with a 10-second interval. The measurement was conducted under 630 nm red light illumination at an intensity of 6 mW/cm² and 0V bias. The results indicate that the photocurrent remains stable, with the device consistently exhibiting strong photoresponse throughout the repeated switching cycles.

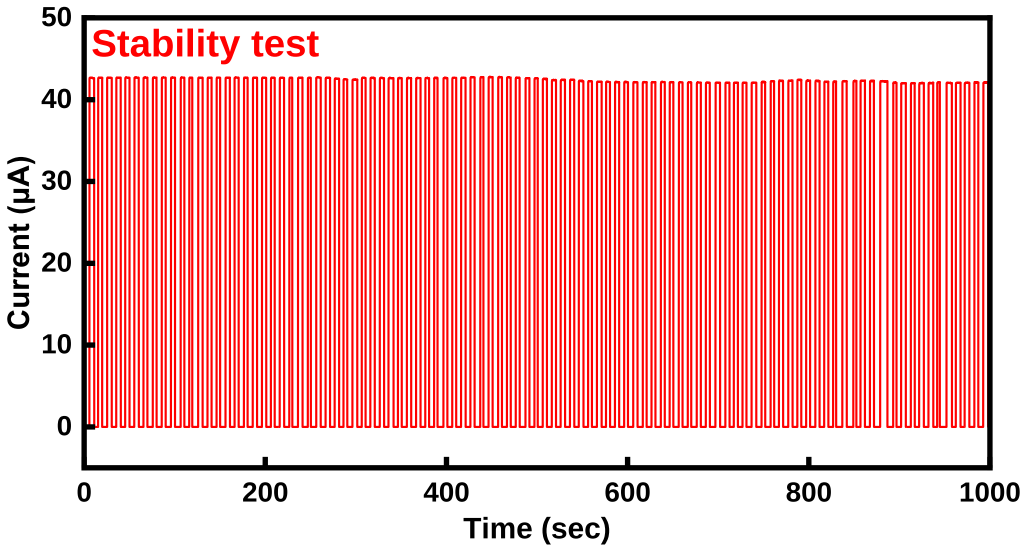

Figure S5. Stability test in 1000 sec period

Figure S6 shows the comparison of dark current with and without 3nm SiO_2 in the CdS devices. After introducing a 3 nm-thick SiO_2 layer into the CdS-based device, a noticeable reduction in dark current was observed, decreasing from 80 nA to 50 nA. However, this improvement still falls short of the significantly lower dark current observed in Bi_2O_2Se -based devices. This discrepancy may be attributed to differences in interfacial properties and the intrinsic electrical characteristics of the two materials. As a result, even under identical passivation conditions, the two systems exhibit distinct responses, and the optimal thickness of the passivation layer may differ accordingly. These findings underscore the importance of material-dependent interface engineering

in effectively suppressing dark current.

The reason we did not directly compare Bi_2O_2Se-based devices with and without the SiO_2 layer is that the PLD process involves elevated temperatures and the introduction of oxygen. Even when Bi_2O_2Se is grown immediately after substrate cleaning, spontaneous formation of SiO_2 on the silicon surface is inevitable during the deposition process. Compared to our intentionally treated Si substrates, this native oxide is more difficult to control in terms of quality and thickness. In contrast, the treated Si substrates with native oxide serve as a reliable and consistent protective layer, allowing for better control of experimental variables.

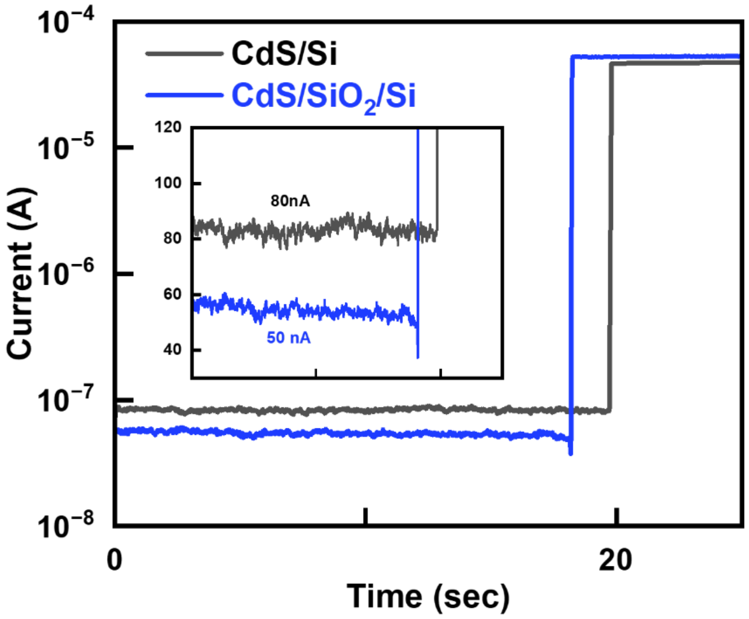

Figure S6. Comparison of dark current with and without 3nm SiO_2 in the CdS devices

A 13 nm Bi_2O_2Se/3 nm SiO_2/p-Si heterostructure demonstrated optimal performance with a high responsivity of 23.0 A/W, ultrafast response (<1 ms), and extremely low dark current (~7 pA). The introduction of a tunneling SiO_2 layer

minimized dark current while maintaining high photocurrent, verified by WKB-based tunneling probability analysis. Device reproducibility and stability were confirmed (Figures S4–S5), and comparison with CdS/Si structures (Figures 5d–f, S6, and Table S2) further highlighted the superior low-dark-current and fast-response performance of Bi_2O_2Se-based devices due to its high mobility, anisotropic conductivity, and effective passivation.

Table S2. Detailed experimental data comparison of **Figures 4** and **5**

Materials	Conditions	R ($A{\cdot}W^{-1}$)	Time (Rise/Decay)	Dark Current (pA)	On/Off
$Bi_2O_2Se/SiO_2/Si$	630nm/0V	23.0	<1ms	7	7.9×10^6
$Bi_2O_2Se/SiO_2/Si$	532nm/0V	17.9	<1ms	7	3.0×10^6
$Bi_2O_2Se/SiO_2/Si$	350nm/0V	0.045	<1ms	7	1.1×10^4
CdS/Si	630nm/0V	26.0	~11ms	8×10^4	6.3×10^2
$CdS/SiO_2/Si$	630nm/0V	26.0	~11ms	5×10^4	1.0×10^3

Reference

(1) Ling, D.; Wang, Q.; Tian, G.; Yu, H.; Zhang, D.; Wang, Q. Oxygen vacancy-enriched Bi2SeO5 nanosheets with dual mechanism for ammonium-ion batteries. ACS nano 2023, 17 (24), 25222-25233.

(2) Huang, X.; Niu, C.-Y.; Zhang, J.; Wang, A.; Jia, Y.; Song, Y. Strain-tunable electronic structure, optical response, and high electron mobility of Bi_2O_2Se crystals. APL Materials 2019, 7 (8), 081110.
